# Impact of Baseline Corticosteroid Use on the Efficacy and Safety of Upadacitinib in Patients with Ulcerative Colitis: A Post Hoc Analysis of the Phase 3 Clinical Trial Programme

**DOI:** 10.1093/ecco-jcc/jjad190

**Published:** 2023-11-06

**Authors:** Tim Raine, Yoh Ishiguro, David T Rubin, Tricia Finney-Hayward, Ramona Vladea, John Liu, Charles Phillips, Erica Cheng, Laura Targownik, Edward V Loftus

**Affiliations:** Department of Gastroenterology, Addenbrooke’s Hospital, Cambridge University Hospitals, Cambridge, UK; Department of Clinical Research, Hirosaki General Medical Centre, National Hospital Organisation, Hirosaki, Japan; Medicine Inflammatory Bowel Disease Center, University of Chicago, Chicago, IL, USA; Former employee of AbbVie, Global Medical Affairs, Maidenhead, UK; AbbVie, Global Medical Affairs, North Chicago, IL, USA; AbbVie, Research and Development, North Chicago, IL, USA; AbbVie, Research and Development, North Chicago, IL, USA; AbbVie, Data and Statistical Sciences, North Chicago, IL, USA; Division of Gastroenterology and Hepatology, Mount Sinai Hospital, University of Toronto, Toronto, ON, Canada; Division of Gastroenterology and Hepatology, Mayo Clinic College of Medicine and Science, Rochester, MN, USA

**Keywords:** Clinical trials

## Abstract

**Background and Aims:**

This post hoc analysis assessed the efficacy and safety of upadacitinib in patients with moderately to severely active ulcerative colitis stratified by corticosteroid use from the ulcerative colitis Phase 3 clinical trial programme.

**Methods:**

Patients were randomised [1:2] to 8 weeks’ placebo or upadacitinib 45 mg once daily; Week 8 responders were re-randomised [1:1:1] to 52 weeks’ placebo or upadacitinib 15 or 30 mg daily. Corticosteroid dose was kept stable during induction but tapered according to a protocol-defined schedule [or investigator discretion] during maintenance Weeks 0–8. Efficacy outcomes and exposure-adjusted, treatment-emergent adverse event [TEAE] rates were assessed for induction and maintenance stratified by corticosteroid use at induction baseline.

**Results:**

Overall, 377/988 [38%] patients were receiving corticosteroids at induction baseline [placebo, *n *= 133; upadacitinib 45 mg, *n* = 244] and 252 [37%] of the 681 clinical responders who entered maintenance were on corticosteroids at induction baseline [*n *= 84 for each treatment]. Similar proportions of patients receiving upadacitinib achieved clinical remission per Adapted Mayo Score with and without baseline corticosteroids at Weeks 8 and 52. The total proportion of patients re-initiating corticosteroids was higher with placebo [24/84;29%] vs upadacitinib 15 mg [16/81; 20%)] and 30 mg [11/81; 14%]. During induction, patients receiving corticosteroids at baseline had higher rates of TEAEs, serious TEAEs, and serious infections vs those not receiving corticosteroids; however, TEAE rates were similar during maintenance after corticosteroid withdrawal.

**Conclusions:**

Upadacitinib is an effective steroid-sparing treatment in patients with moderately to severely active ulcerative colitis. Clinicaltrials.gov identifiers: NCT02819635; NCT03653026

## 1. Introduction

Corticosteroids [CS] are often used to treat flare-related symptoms in patients with ulcerative colitis [UC]. They act rapidly, making them a particularly effective intervention when desiring rapid improvement of UC symptoms.^1,2^ However, CS are not effective in promoting mucosal healing, thereby making them ineffective for maintenance therapy.^3^ In addition, long-term CS exposure for inflammatory bowel disease [IBD] can be associated with numerous irreversible side effects, including osteoporosis, fractures, and glaucoma,^4^ as well as increased mortality rates and risk of serious and opportunistic infections, compared with tumour necrosis factor inhibitors.^5^ The psychological burden of UC for patients can also be exacerbated by the use of CS through the impact of non-volitional weight gain, sleep and mood disturbance, and, of particular note, suicidal thoughts or attempts.^6^

Owing to these safety concerns, the 2021 European Crohn’s and Colitis Organisation guidelines on therapeutics in UC recommend that courses of CS should be limited to a maximum of 3 months, and CS-sparing agents should be considered in patients requiring more than one course of systemic CS in a year or who have CS-refractory disease.^7^ However, despite these recommendations, studies estimate that, of approximately 30% of patients with IBD who receive CS, 15–20% of these have CS excess or dependency; this proportion is even more pronounced in patients with moderately to severely active UC.^8,9^ As such, although CS play an important role in the short-term treatment of UC, overuse, risk of dependency, and long-term negative effects mean that efficacious CS-sparing agents are recommended.

Upadacitinib [UPA], an oral, selective Janus kinase [JAK] inhibitor, is approved for the treatment of moderately to severely active UC.^10,11^ Approval was based on demonstration of efficacy compared with placebo [PBO], as well as an acceptable safety profile, in a Phase 3 clinical trial programme comprising two identical induction trials of UPA 45 mg once daily [QD; U-ACHIEVE Induction (NCT02819635) and U-ACCOMPLISH (NCT03653026)], and a maintenance study of UPA 15 mg QD and UPA 30 mg QD [U-ACHIEVE Maintenance].^12^

In clinical trials, CS are generally either withdrawn or administered at a fixed dose during the induction period. However, it remains unclear how fixing the CS dose for induction affects the efficacy and/or safety of the study drug being evaluated. Therefore, the aim of this post hoc analysis of patients from the UPA Phase 3 clinical trial programme for UC was to compare the efficacy and safety of 8 weeks’ induction therapy, and subsequent 52 weeks’ maintenance therapy, with UPA in patients with and without CS use, at the start of induction treatment. These aims also enable the exploration of the CS-sparing effect of UPA throughout the maintenance treatment period.

## 2. Materials and Methods

### 2.1. Study design and patients

**Figure 1.**
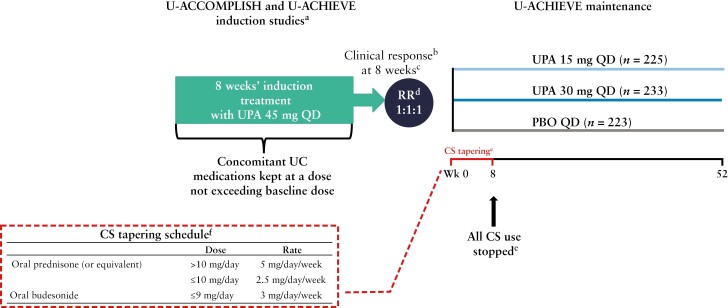
Study designs. Data are for the ITT population, pooled for the U-ACCOMPLISH and U-ACHIEVE Induction studies, and defined as patients who were randomised and received at least one dose of study drug [PBO or UPA 45 mg QD] during the 8-week induction period. For maintenance, this was defined as UPA 45 mg QD 8-week induction responders who were enrolled per protocol for the 52-week maintenance treatment period and received at least one dose of study drug [PBO, UPA 15 mg QD, or UPA 30 mg QD]. ^a^Initial randomisation was stratified by baseline bio-IR status [yes or no], corticosteroid use [yes or no], and Adapted Mayo Score [≤7 or >7]. Baseline was defined as the last non-missing value collected on or before the first dose in the two induction studies. ^b^Clinical response was defined as a decrease in Adapted Mayo Score of ≥2 points and ≥30% from baseline, plus a decrease in rectal bleeding sub-score of ≥1 or an absolute rectal bleeding sub-score of ≤1. ^c^21 patients entered from the Phase 2b study.^17^^d^RR was stratified by bio-IR status [bio-IR or non-bio-IR], CS use at Week 0 of maintenance [yes or no], and clinical remission status at Week 0 of maintenance [yes or no]. ^e^All CS use was stopped by maintenance Week 8 and tapered according to the protocol-defined schedule shown in the table [except at the discretion of the investigator]; however, if a patient had worsening of UC during or after the initial CS taper, CS could be re-initiated, or the dose increased, at the investigator’s discretion. ^f^Oral budesonide-MMX [eg, Cortiment, eUceris] and oral beclomethasone were discontinued. Bio-IR, biologic inadequate response [defined as inadequate response, loss of response, or intolerance to one or more biologic]; CS, corticosteroids; ITT, intention to treat; MMX, multimatrix; PBO, placebo; QD, once daily; RR, re-randomisation; UC, ulcerative colitis; UPA, upadacitinib; Wk, Week.

The full details of the U-ACCOMPLISH, U-ACHIEVE Induction, and U-ACHIEVE Maintenance studies have been reported previously.^12,13^ Briefly, in the induction trials, patients were randomised 2:1 to UPA 45 mg QD or PBO for 8 weeks’ induction therapy. Patients who achieved a clinical response per Adapted Mayo Score [Full Mayo Score excluding Physician Global Assessment] at Week 8 in these studies and the Phase 2b dose-ranging study, were re-randomised 1:1:1 to PBO,^14^ UPA 15 mg QD, or UPA 30 mg QD for 52 weeks’ maintenance therapy in U-ACHIEVE Maintenance [Figure 1]. Patients who were initially randomised to PBO but did not achieve a clinical response at Week 8 were treated with open-label UPA 45 mg QD for 8 weeks; responders after 8 weeks’ open-label UPA induction therapy were also re-randomised into the maintenance study. Patients were not randomised before induction or re-randomised before maintenance according to CS use, but were stratified by CS use at induction baseline [Week 0 of induction] and maintenance baseline [Week 0 of maintenance]. Stratification was performed according to any CS use [regardless of indication], rather than use explicitly reported for treatment of UC.

During the 8-week induction studies, concomitant UC medications [including oral CS, antibiotics, aminosalicylates, and/or methotrexate] were kept at stable doses. Only oral CS use was permitted; the use of CS intravenously or by enemas/suppositories [other than required for endoscopy] was not permitted within 14 days before screening, during the remainder of the screening period, and during the study [induction and maintenance]. In addition, patients were not eligible if they were receiving: [i] an oral CS dose of >30 mg/day [prednisone or equivalent]; [ii] an oral budesonide dose of >9 mg/day; [iii] an oral beclomethasone dose of >5 mg/day or had not been on the current course for at least 14 days before induction baseline [Week 0 induction] and on a stable dose for at least 7 days before induction baseline; or [iv] both oral budesonide [or oral beclomethasone] and oral prednisone [or equivalent] simultaneously, with the exception of topical treatment or inhalers, within 14 days before screening or during the screening period. Following the 8-week induction period, patients who were receiving CS therapy had their dose tapered according to a protocol-defined schedule [shown in Figure 1] or at the discretion of the investigator.

All CS use was stopped by maintenance Week 8 [except at the discretion of the investigator]; however, if a patient had worsening of UC during or after the initial CS taper, CS could be re-initiated, or the dose increased, at the investigator’s discretion. Patients in whom the maximum CS dose exceeded their dose at induction or maintenance baseline were considered non-responders for efficacy assessments from that time point until the end of that study. These patients were still assessed for safety.

In this post hoc analysis, efficacy and safety outcomes during the 8-week induction and 52-week maintenance periods were analysed by CS use at induction baseline.

### 2.2. Quantification of CS dose

The sum of daily dose of CS was evaluated at induction baseline [Week 0 induction] by converting each CS dose to prednisone-equivalent doses. CS use for non-UC indications was excluded.

### 2.3. Efficacy endpoints by CS use at induction baseline

The proportion of patients achieving clinical remission [Adapted Mayo Score ≤2, with stool frequency sub-score ≤1 and not greater than induction baseline, rectal bleeding score = 0, and endoscopic sub-score ≤1 without friability] was assessed at Week 8 [induction] and at Week 52 [maintenance] in patients with and without CS use at induction baseline. Partial Mayo Score [Full Mayo Score excluding endoscopy sub-score] was assessed at baseline and every 2 weeks thereafter in the induction studies, and at Weeks 4, 8, 12, and every 8 weeks thereafter in the maintenance study in patients with and without CS use at induction baseline. Partial Mayo Score was used to assess disease activity over time, because it was measured at numerous time points throughout the induction and maintenance trials, whereas other measures [eg, Full Mayo Score and Adapted Mayo Score] contain endoscopic sub-scores, which were only measured at induction baseline [Week 0], Week 8 of induction, and Week 52 of maintenance. The proportion of patients achieving clinical remission per Adapted Mayo Score and who were CS-free for 90 or 183 days immediately before Week 52, among patients with and without CS use at induction baseline, were evaluated and compared with those achieving clinical remission per Adapted Mayo Score at Week 52 without CS use at induction baseline.

### 2.4. CS re-initiation and cumulative dose during maintenance

The proportion of patients who re-initiated CS during maintenance was assessed, defined as patients who were receiving CS at any visit during maintenance after initial discontinuation. In addition, the proportion of patients with CS use at baseline who were CS-free at each assessed time point during maintenance [Weeks 8, 12, 20, 28, 36, 44, and 52 of maintenance] was also assessed.

Among all patients with CS use during maintenance [regardless of CS use at induction baseline], the cumulative CS dose [prednisone equivalent] during the maintenance period was calculated by summing the CS doses at Weeks 0, 4, 8, 12, 20, 28, 36, 44, and 52. A second analysis was conducted to assess CS use after the taper [summing CS doses at Weeks 12, 20, 28, 36, 44, and 52].

### 2.5. Treatment-emergent adverse events by CS use at induction baseline

Treatment-emergent adverse events [TEAEs] and TEAEs of special interest were collected in the induction and maintenance studies. TEAEs of special interest were pre-specified based on previous findings in patients treated with UPA for other immune-mediated indications, or other JAK inhibitors in UC,^15,16^ eg, serious infections, opportunistic infections, herpes zoster, malignancy excluding non-melanoma skin cancer [NMSC], NMSC, gastrointestinal [GI] perforations, major adverse cardiovascular events [MACEs], and venous thromboembolic events [VTEs]. MACEs and VTEs were adjudicated by an independent external adjudication committee, and GI perforation by an independent internal committee.

### 2.6. Statistical analysis

Four populations were assessed: [i] induction intention to treat [ITT; patients who were randomised and received at least one dose of study drug (PBO or UPA 45 mg QD) during the 8-week induction period] for induction efficacy analyses; [ii] maintenance ITT [UPA 45 mg QD 8-week induction responders who were enrolled per protocol for the 52-week maintenance treatment period and received at least one dose of study drug (PBO, UPA 15 mg QD, or UPA 30 mg QD)] for maintenance efficacy analyses; [iii] induction safety [all patients who were randomised and received at least one dose of study drug (PBO or UPA 45 mg QD) during the 8-week induction period]; and [iv] maintenance safety [all UPA 45 mg QD 8-week induction responders who were enrolled per protocol for 52-week maintenance therapy and received at least one dose of study drug (PBO, UPA 15 mg QD, or UPA 30 mg QD)].

Clinical remission at Week 8 of induction and Week 52 of maintenance by baseline CS use was analysed using the Cochran–Mantel–Haenszel test adjusted for strata [induction: stratified by Adapted Mayo Score at baseline (≤7 or >7) and biologic inadequate responder (bio-IR) status (defined as inadequate response, loss of response, or intolerance to one or more biologic; bio-IR or non-bio-IR); maintenance: stratified by bio-IR status [bio-IR or non-bio-IR] and clinical remission status at week 0 of maintenance (yes or no)]. CS-free clinical remission was analysed in the same way, but CS use at Week 0 of maintenance [yes or no] was included in the Cochran–Mantel–Haenszel test as a stratum. Non-responder imputation incorporating multiple imputation was for missing data, with multiple imputation used to handle missing data due to COVID-19, and non-responder imputation used for all other missing data.

The differences in Partial Mayo Score over time by baseline CS use were analysed using a mixed-effects model with repeated measurement regression, with baseline CS use as a stratification criterion, baseline Partial Mayo Score as a continuous covariate, and the following as fixed factors within each subgroup: treatment, visit, treatment by visit interaction, and strata.

In the primary efficacy analyses, patients with worsening of UC who initiated, re-initiated, or dose-increased CS after the maintenance taper, above their dose at baseline, were considered non-responders for efficacy assessments from that time point until the end of the study. To assess the impact of CS use on efficacy during maintenance, sensitivity analyses were performed for the proportion of patients in clinical remission per Adapted Mayo Score at Week 52, in which any patient who received CS after Week 8 of maintenance was considered a non-responder [regardless of dose], as well as Partial Mayo Score over time from Week 0 to Week 52 of maintenance, in which if a patient received CS after Week 8, measurements were excluded for that patient from the CS start date onwards.

All efficacy analyses were post hoc, meaning that endpoints were not multiplicity controlled and all *p*-values are nominal.

Rates of TEAEs are presented as exposure-adjusted event rates [EAERs; events/100 patient-years (E/100 PYs)] with 95% confidence intervals [CIs], as well as exposure-adjusted incidence rates (EAIRs; number of patients/100 PYs (*n*/100 PYs)] for adverse events of special interest [AESIs]. PYs for EAERs were calculated from the first dose of study drug received during induction through to the last dose received. PYs for EAIRs were calculated from the first dose of study drug through to the time of first TEAE occurrence.

### 2.7. Ethics statement

The U-ACCOMPLISH and U-ACHIEVE Induction studies, and U-ACHIEVE Maintenance study were conducted in accordance with the International Conference on Harmonization guidelines, applicable regulations, and the Declaration of Helsinki. Study‐related documents were approved by the relevant ethics committees/institutional review boards. All patients provided written informed consent before screening.

## 3. Results

### 3.1. Patient demographics and disease characteristics

**Figure 2.**
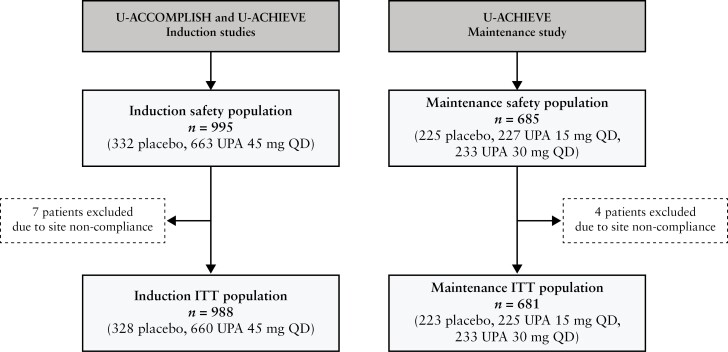
Population flow diagram. ITT, intention to treat; QD, once daily; UPA, upadacitinib.

Overall, 995 patients [PBO, *n* = 332; UPA 45 mg QD, *n* = 663] received at least one dose of study drug during the 8-week induction period and comprised the induction safety population. Of these, 988 patients were included in the induction ITT population [PBO, *n* = 328; UPA 45 mg QD, *n* = 660]; seven patients were excluded due to site non-compliance [Figure 2]. In the ITT population, 377 [38.2%] were receiving CS at induction baseline [PBO, *n* = 133; UPA 45 mg QD, *n *= 244].

For maintenance, 685 patients [PBO, *n *= 225; UPA 15 mg QD, *n* = 227; UPA 30 mg QD, *n* = 233] who achieved a clinical response after 8 weeks’ induction therapy with UPA 45 mg QD [including those who initially received 8-week double-blind PBO but were subsequently treated with 8 weeks’ open-label UPA 45 mg QD, and 21 patients from the Phase 2b study]^17^ were re-randomised into the maintenance study and received at least one dose of study drug during this period, and comprise the maintenance safety populations [Figure 2]. The ITT population included 681 patients [PBO, *n* = 223; UPA 15 mg QD, *n* = 225; UPA 30 mg QD, *n* = 233]; the difference between the safety and ITT populations was because four patients [two each from the PBO and UPA 15 mg QD groups] were excluded from the ITT population efficacy analyses due to site non-compliance, but these patients were still assessed for safety. In the ITT population, 252 [37.0%] were receiving CS at induction baseline [*n* = 84 each for UPA 15 mg, UPA 30 mg, and PBO].

Patient demographics and disease characteristics in the induction and maintenance ITT populations assessed at induction baseline were generally well balanced between patients who were and were not receiving CS at induction baseline, except for numerically higher proportions of bio-IR patients and patients with extensive disease/pancolitis seen in patients with baseline CS use [Table 1].

**Table 1.** Baseline demographics and clinical characteristics by CS use at induction baseline [ITT population].

	Pooled U-ACCOMPLISH and U-ACHIEVE Induction				U-ACHIEVE Maintenance					
	No CS use at induction baseline [*n *= 611]		CS use at induction baseline [*n *= 377]		No CS use at induction baseline [*n* = 429]			CS use at induction baseline [*n* = 252]		
	Placebo [*n *= 195]	UPA 45 mg QD [*n* = 416]	Placebo [*n* = 133]	UPA 45 mg QD [*n* = 244]	Placebo [*n* = 139]	UPA 15 mg QD [*n *= 141]	UPA 30 mg QD [*n* = 149]	Placebo [*n* = 84]	UPA 15 mg QD [*n* = 84]	UPA 30 mg QD [*n *= 84]
Female	71 [36.4]	149 [35.8]	53 [39.8]	99 [40.6]	63 [45.3]	47 [33.3]	56 [37.6]	37 [44.0]	32 [38.1]	36 [42.9]
Age, years, mean [SD]	44.8 [15.3]	43.2 [14.6]	40.9 [13.0]	42.2 [14.2]	42.3 [15.0]	42.4 [15.1]	42.7 [14.6]	42.7 [13.7]	40.5 [12.6]	43.5 [14.7]
Weight, kg, mean [SD]	74.0 [17.8]	73.5 [18.3]	73.6 [22.1]	71.2 [17.6]	72.4 [18.1]	73.3 [19.7]	73.0 [18.1]	71.6 [18.4]	71.7 [19.7]	72.6 [20.7]
Disease duration, years, mean [SD]	8.4 [8.3]	7.9 [6.8]	8.0 [7.6]	8.1 [6.9]	8.8 [8.1]	7.6 [7.6]	8.2 [6.9]	7.8 [7.2]	9.2 [6.6]	7.5 [7.0]
UC extent										
Left-sided	101 [51.8]	213 [51.2]	61 [45.9]	109 [44.7]	76 [54.7]	68 [48.2]	73 [49.0]	41 [48.8]	34 [40.5]	33 [39.3]
Extensive/pancolitis	94 [48.2]	202 [48.6]	72 [54.1]	135 [55.3]	63 [45.3]	73 [51.8]	76 [51.0]	43 [51.2]	50 [59.5]	51 [60.7]
Aminosalicylates use	136 [69.7]	298 [71.6]	87 [65.4]	155 [63.5]	96 [69.1]	101 [71.6]	109 [73.2]	50 [59.5]	59 [70.2]	59 [70.2]
Prior anti-TNF use	80 [41.0]	176 [42.3]	76 [57.1]	150 [61.5]	57 [41.0]	55 [39.0]	56 [37.6]	50 [59.5]	49 [58.3]	51 [60.7]
Bio-IR status										
Bio-IR	86 [44.1]	185 [44.5]	81 [60.9]	155 [63.5]	62 [44.6]	58 [41.1]	59 [39.6]	54 [64.3]	51 [60.7]	52 [61.9]
Non-bio-IR	109 [55.9]	231 [55.5]	52 [39.1]	89 [36.5]	77 [55.4]	83 [58.9]	90 [60.4]	30 [35.7]	33 [39.3]	32 [38.1]
Partial Mayo Score, mean [SD]	6.7 [1.4]	6.6 [1.4]	6.6 [1.3]	6.6 [1.4]	6.8 [1.4]	6.5 [1.3]	6.7 [1.3]	6.4 [1.2]	6.5 [1.5]	6.7 [1.4]
Adapted Mayo Score, mean [SD]	7.0 [1.3]	7.0 [1.2]	7.0 [1.1]	7.0 [1.2]	7.1 [1.2]	6.9 [1.2]	7.0 [1.3]	6.8 [1.2]	6.9 [1.3]	7.1 [1.2]
hs-CRP, mg/L, median [range]	4.7 [0.2–143.0]	4.4 [0.0–107.0]	4.4 [0.2–179.0]	3.2 [0.2–96.8]	3.9 [0.2–105.0]	4.3 [0.2–67.9]	4.1 [0.2–107.0]	3.9 [0.2–84.7]	2.4 [0.2–83.3]	4.2 [0.2–96.8]
Faecal calprotectin, mg/kg, median [range]	*n* = 171 1753.0 [30.0–28,800.0]	*n* = 359 1705.0 [30.0–28,800.0]	*n* = 118 1783.0 [84.0–28,129.0]	*n *= 214 1752.0 [30.0–28,800.0]	*n *= 122 1827.0 [30.0–28,800.0]	*n* = 117 1557.0 [30.0–28,800.0]	*n *= 126 1550.0 [75.0–28,800.0]	*n* = 73 1357.0 [30.0–28,800.0]	*n* = 80 1918.5 [77.0–26,911.0]	*n* = 67 1651.0 [30.0–15,983.0]

Data are *n* [%] unless otherwise specified. Data are for the ITT population, pooled for the U-ACCOMPLISH and U-ACHIEVE Induction studies, and defined as patients who were randomised and received at least one dose of study drug [placebo or UPA 45 mg QD] during the 8-week induction period. For maintenance, this was defined as UPA 45 mg QD 8-week induction responders who were enrolled per protocol for the 52-week maintenance treatment period and received at least one dose of study drug [placebo, UPA 15 mg QD, or UPA 30 mg QD].

Bio-IR, biologic inadequate response [defined as inadequate response, loss of response, or intolerance to one or more biologic]; CS, corticosteroids; hs-CRP, high-sensitivity C-reactive protein; ITT, intention to treat; QD, once daily; SD, standard deviation; TNF, tumour necrosis factor; UC, ulcerative colitis; UPA, upadacitinib.

#### 3.1.1. Proportion of patients who tapered CS by maintenance Week 8

Most patients successfully tapered according to the protocol-defined tapering regimen [139/246 (56.5%)], with more patients successfully tapering with UPA 30 mg QD [55/81 (67.9%)] and UPA 15 mg QD [49/81 (60.5%)] vs PBO [35/84 (41.7%)].

### 3.2. Quantification of CS dose

Among the maintenance ITT population with CS use [*n* = 252], the median total daily CS dose at induction baseline [Week 0] was similar for patients randomised to PBO, UPA 15 mg QD, or UPA 30 mg QD [Table 2]. Most patients were on ≤25 mg prednisone equivalent in the PBO [80.7%], UPA 15 mg QD [69.1%], and UPA 30 mg QD [82.7%] groups. These analyses should be interpreted with respect to the fact that two patients [PBO, *n *= 1; UPA 15 mg QD, *n* = 1] had reported baseline CS doses substantially greater than the usual maximum administered daily dose [>80 mg of prednisone or equivalent].

**Table 2.** Quantification of CS dose at induction baseline [Week 0] among the maintenance ITT population [*n* = 252].^a^

Prednisone equivalent daily dose of CS, mg	PBO [*n* = 83]	UPA 15 mg QD [*n* = 81]	UPA 30 mg QD [*n* = 81]
Mean [SD]	199 [1649]	576 [4998]	19 [10]
Median [IQR]	20 [10–25]	15 [10–30]	20 [10–20]
Dose categories, *n* [%]			
>0–≤2.5 mg	1 [1.2]	2 [2.5]	0
>2.5–≤5 mg	10 [12.0]	8 [9.9]	5 [6.2]
>5–≤25 mg	56 [67.5]	46 [56.8]	62 [76.5]
>25–≤30 mg	8 [9.6]	12 [14.8]	7 [8.6]
>30–≤50 mg^b^	7 [8.4]	12 [14.8]	7 [8.6]
>50 mg^b^^,^^c^	1 [1.2]	1 [1.2]	0

^a^Comprises patients who were UPA 45 mg QD 8-week induction responders and who were enrolled under the protocol for the 52-week maintenance treatment period in Cohort 1 and received at least one dose of study drug in the maintenance study.

^b^Patients could be enrolled into the induction trials in violation of the protocol-defined exclusion criterion of a CS dose limit of 30 mg prednisone equivalent on a case-by-case basis; continuation in the trials was decided by the Therapeutic Area Medical Director.

^c^These patients had reported doses substantially greater than the usual maximum administered dose [>80 mg of prednisone or equivalent].

CS, corticosteroids; IQR, interquartile range; ITT, intention to treat; PBO, placebo; QD, once daily; SD, standard deviation; UPA, upadacitinib.

### 3.3. Efficacy measures

#### 3.3.1. Patients in clinical remission at Week 8 of induction and Week 52 of maintenance by CS use at induction baseline

**Figure 3.**
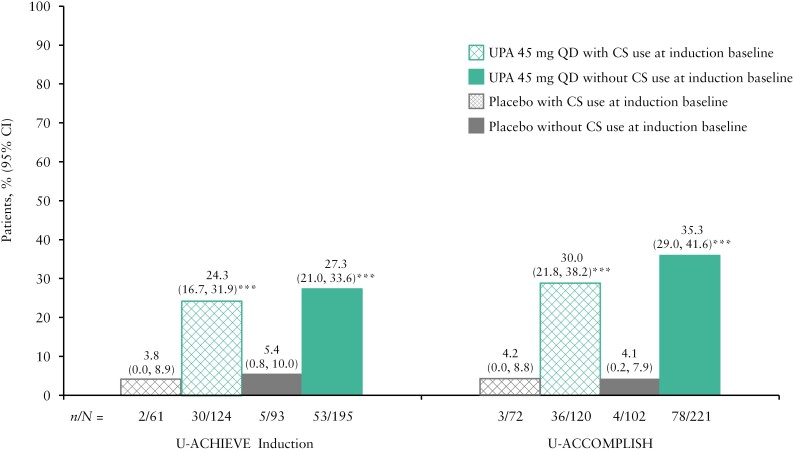
Proportion of patients in clinical remission per Adapted Mayo Score at the end of induction [Week 8], by CS use at induction baseline. Data are for the ITT population, pooled for U-ACCOMPLISH and U-ACHIEVE Induction studies, and defined as patients who were randomised and received at least one dose of study drug [placebo or UPA 45 mg QD] during the 8-week induction period. Clinical remission per Adapted Mayo Score was defined as Adapted Mayo Score ≤2, stool frequency score ≤1 and not greater than baseline, rectal bleeding sub-score = 0, and endoscopic sub-score ≤1. ***Nominal *p* <0.001 vs placebo. CI, confidence interval; CS, corticosteroids; ITT, intention to treat; QD, once daily; UPA, upadacitinib.

A higher proportion of patients receiving UPA 45 mg QD achieved clinical remission per Adapted Mayo Score at Week 8 of induction compared with PBO, regardless of CS use [all nominal *p* <0.001; Figure 3]. Similar proportions of patients receiving UPA 45 mg QD with and without CS use at induction baseline achieved this endpoint [Figure 3].

**Figure 4.**
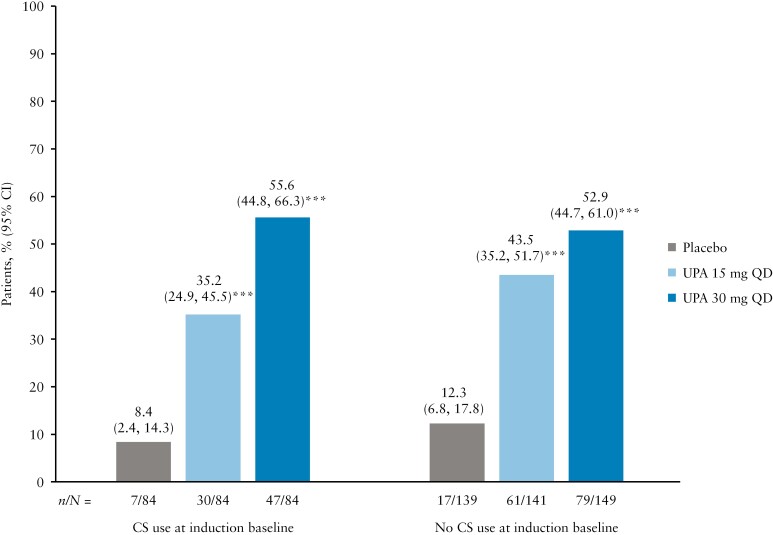
Proportion of patients in clinical remission per Adapted Mayo Score at the end of maintenance [Week 52], by CS use at induction baseline. Data are for the ITT population. For maintenance, this was defined as UPA 45 mg QD 8-week induction responders who were enrolled per protocol for the 52-week maintenance treatment period and received at least one dose of study drug. Clinical remission per Adapted Mayo Score was defined as Adapted Mayo Score ≤2, stool frequency score ≤1 and not greater than baseline, rectal bleeding sub-score = 0, and endoscopic sub-score ≤1. ***Nominal *p* <0.001 vs placebo. CI, confidence interval; CS, corticosteroids; ITT, intention to treat; QD, once daily; UPA, upadacitinib.

Similarly, at Week 52 of maintenance, a higher proportion of patients achieved clinical remission with both UPA doses compared with PBO, regardless of CS use at induction baseline [all nominal *p* <0.001; Figure 4]. The proportions of patients with and without CS use at induction baseline achieving clinical remission per Adapted Mayo Score were similar, regardless of treatment [8.4%/35.2%/55.6% for PBO, UPA 15 mg QD, and UPA 30 mg QD, respectively, with CS use, compared with 12.3%/43.5%/52.9%, respectively, without CS use; Figure 4]. The results of the sensitivity analysis for maintenance, in which any patient who received CS after Week 8 of maintenance was considered a non-responder, aligned with the main analysis; a higher proportion of patients achieved clinical remission with both UPA doses compared with PBO, regardless of CS use at induction baseline [all nominal *p* <0.001; Supplementary Figure 1].

#### 3.3.2. Partial Mayo Score throughout maintenance by CS use at induction baseline

**Figure 5.**
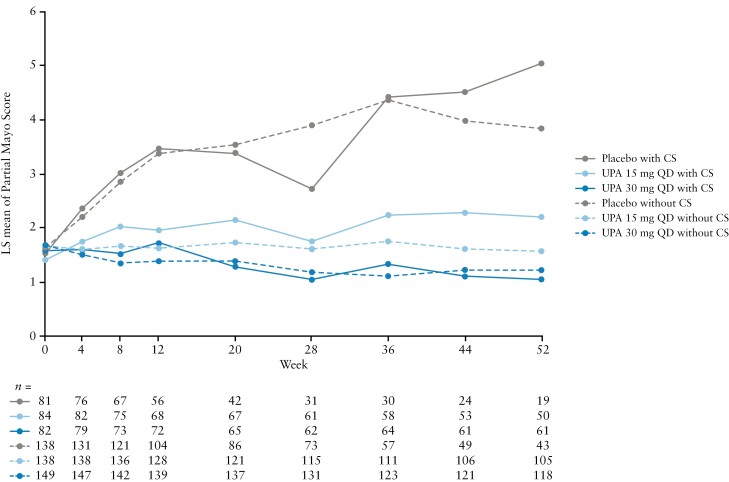
Partial Mayo Score from Week 0 to Week 52 of maintenance in patients with or without CS use at induction baseline. Data are for the ITT population. For maintenance, this was defined as UPA 45 mg QD 8-week induction responders who were enrolled per protocol for the 52-week maintenance treatment period and received at least one dose of study drug [placebo, UPA 15 mg QD, or UPA 30 mg QD]. CS, corticosteroids; ITT, intention to treat; LS, least squares; QD, once daily; UPA, upadacitinib.

Partial Mayo Scores were numerically lower in both UPA groups compared with the PBO group by Week 4 of maintenance for patients with and without CS use at induction baseline, and remained numerically lower through Week 52 [Figure 5]. In patients with CS use at induction baseline, Partial Mayo Scores with PBO and both UPA doses were generally numerically higher throughout maintenance compared with patients with no CS use at induction baseline [Figure 5]. The differences in Partial Mayo Score between the UPA 15 mg QD and UPA 30 mg QD doses were generally more pronounced in patients with vs without CS at induction baseline throughout maintenance, particularly from around Week 20 onwards. The results of the sensitivity analysis for maintenance, in which if a patient received CS after Week 8, measurements were excluded for that patient from the CS start date onwards, aligned with the main analysis, with similar patterns in Partial Mayo Scores seen throughout maintenance in patients with and without CS use at induction baseline [Supplementary Figure 2].

#### 3.3.3. Maintenance of CS-free clinical remission per Adapted Mayo Score at Week 52

**Figure 6.**
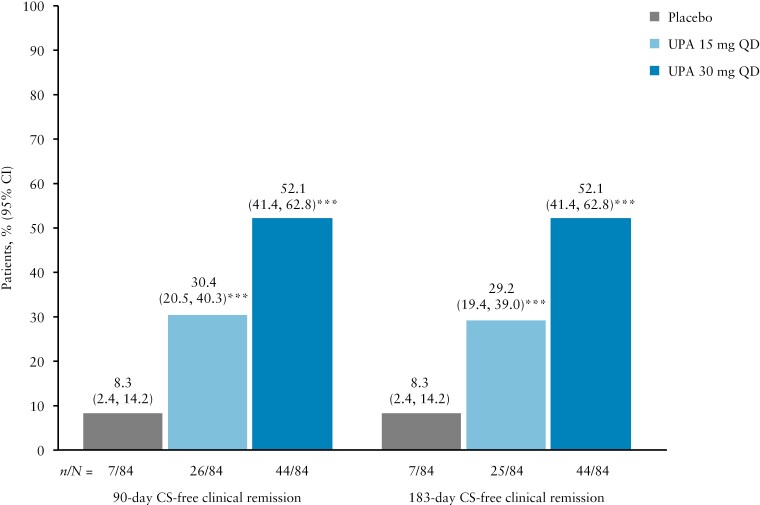
Proportion of patients achieving clinical remission per Adapted Mayo Score and who were CS-free for 90 or 183 days immediately before Week 52, among patients with CS use at induction baseline. Data are for the ITT population. For maintenance, this was defined as UPA 45 mg QD 8-week induction responders who were enrolled per protocol for the 52-week maintenance treatment period and received at least one dose of study drug [placebo, UPA 15 mg QD, or UPA 30 mg QD]. CS-free clinical remission was defined as clinical remission per Adapted Mayo Score [defined as Adapted Mayo Score ≤2, stool frequency score ≤1 and not greater than baseline, rectal bleeding sub-score = 0, and endoscopic sub-score ≤1] at Week 52 and CS-free for ≥90 or ≥183 consecutive days immediately preceding Week 52 among patients taking CS at baseline in the induction study. ***Nominal *p <*0.001 vs placebo. CI, confidence interval; CS, corticosteroids; ITT, intention to treat; QD, once daily; UPA, upadacitinib.

Proportions of patients with CS use at induction baseline achieving 90-day and 183-day CS-free clinical remission at Week 52 were significantly greater with UPA 15 mg QD and UPA 30 mg QD than PBO [all nominal *p* <0.001; Figure 6], with similar results for each CS-free time period. In addition, proportions of patients achieving clinical remission per Adapted Mayo Score and who were CS-free for 90 or 183 days immediately before Week 52 among patients with CS use at induction baseline [30.4% and 29.2%, respectively, for UPA 15 mg QD; 52.1% for both with UPA 30 mg QD] were lower than [15 mg group] or similar to [30 mg group] the proportions of patients achieving clinical remission at Week 52 who had no CS use at induction baseline [43.5% for UPA 15 mg QD and 52.9% for UPA 30 mg QD].

### 3.4. CS re-initiation and cumulative dose during maintenance

**Figure 7.**
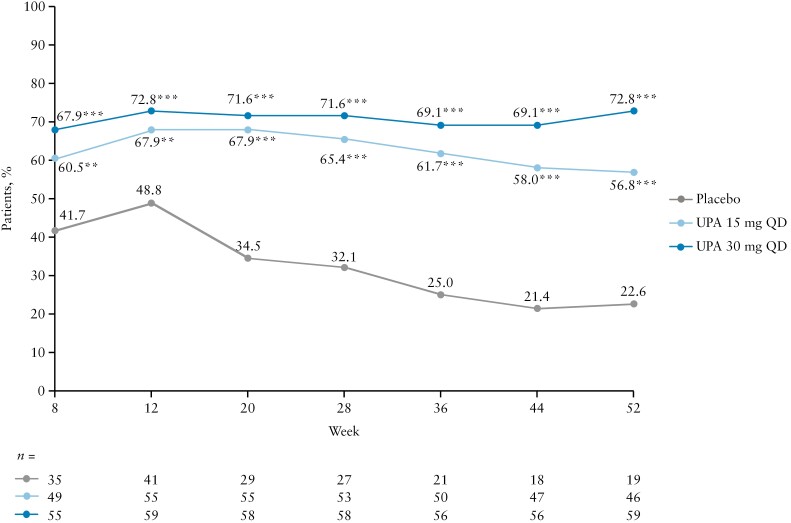
Proportions of patients who were CS-free at each time point during maintenance after the CS taper among patients with CS use at baseline. Data are for the ITT population. For maintenance, this was defined as UPA 45 mg QD 8-week induction responders who were enrolled per protocol for the 52-week maintenance treatment period and received at least one dose of study drug. CS-free clinical remission was defined as clinical remission per Adapted Mayo Score [defined as Adapted Mayo Score ≤2, stool frequency score ≤1 and not greater than baseline, rectal bleeding sub-score = 0, and endoscopic sub-score ≤1] at Week 52 and CS-free for ≥90 or ≥183 consecutive days immediately preceding Week 52 among patients taking CS at baseline in the induction study. **Nominal *p* <0.01 vs placebo. ***Nominal *p* <0.001 vs placebo. CS, corticosteroids; ITT, intention to treat; QD, once daily; UPA, upadacitinib.

Of the patients with CS use at induction baseline, following the CS taper from Week 0 to 8 of maintenance, the total proportion of patients who re-initiated CS was higher with PBO vs UPA 15 and 30 mg QD [28.6% vs 19.8% and 13.6%, respectively]. In addition, the proportions of patients who were CS-free during maintenance after the CS taper at Week 8 were higher at every time point with both UPA doses vs PBO [all nominal *p* <0.001 for both doses, except for Weeks 8 and 12 for UPA 15 mg QD (*p* = 0.005 and *p* = 0.007, respectively); Figure 7]. The magnitude of these differences was particularly pronounced after 12 weeks of CS withdrawal [Week 20 onwards], due to a steady decrease in patients remaining CS-free in the PBO group, whereas the proportions remained consistent in the UPA groups. At each time point, the proportion of CS-free patients was numerically higher with UPA 30 mg QD than UPA 15 mg QD.

Among all patients receiving CS during maintenance [Weeks 0–52], regardless of induction baseline use, the median [interquartile range] cumulative doses were 20 [9, 45] mg, 20 [10, 45] mg, and 23 [10, 45] mg prednisone equivalent for UPA 15 mg QD, UPA 30 mg QD, and PBO, respectively; the mean [standard deviation (SD)] doses were 575.6 [4905.9] mg, 31.9 [34.4] mg, and 1175.8 [10 943.5] mg prednisone equivalent, respectively. When considering cumulative doses after the CS taper period [during Weeks 12–52], the median [interquartile range] cumulative doses were 0 [0–10], 0 [0], and 0 [0], respectively; the mean [SD] doses were 10.5 [34.9] mg, 7.5 [22.0] mg, and 822.7 [6973.5] mg prednisone equivalent for UPA 15 mg QD, UPA 30 mg QD, and PBO, respectively. These analyses should be interpreted with respect to the fact that one patient in the PBO group had a reported CS dose substantially greater than the usual maximum administered daily dose [>80 mg of prednisone or equivalent].

### 3.5. Treatment-emergent AESIs by baseline CS use

At Week 8 of the induction period, EAERs of any TEAE and serious TEAEs were numerically higher in patients with CS use at induction baseline in both the UPA 45 mg QD and PBO groups [Table 3]. In patients receiving UPA 45 mg QD, higher EAERs of serious infection were observed in patients with CS use at induction baseline compared with those without CS use at induction baseline [EAER (95% CI): 13.1 (1.6, 24.6) vs 3.2 (0.0, 7.5) E/100 PYs, respectively]. This pattern was also observed with herpes zoster [5.3 (0.0, 12.5) vs 3.2 (0.0, 7.5) E/100 PYs, respectively]. EAERs of creatine phosphokinase [CPK] elevation and neutropenia were higher in patients receiving UPA 45 mg QD with no baseline CS use compared with patients with baseline CS use [CPK elevation: 42.5 (26.5, 58.6) vs 23.6 (8.2, 39.1) E/100 PYs, respectively; neutropenia: 50.4 (33.0, 67.9) vs 2.6 (0.0, 7.8) E/100 PYs, respectively)]. No malignancy [excluding NMSC] or MACEs were observed. One adjudicated VTE and one adjudicated GI perforation were reported in the PBO without CS use group.

**Table 3.** Overview of TEAEs and AESIs by CS use at induction baseline [safety populations].

	Pooled U-ACCOMPLISH and U-ACHIEVE Induction				U-ACHIEVE Maintenance					
Events [events/100 PYs] [95% CI]	No CS use at induction baseline [*n* = 611]		CS use at induction baseline [*n* = 384]		No CS use at induction baseline [*n* = 429]			CS use at induction baseline [*n* = 256]		
	Placebo [*n* = 195] 28.9 PYs	UPA 45 mg QD [*n* = 416] 63.5 PYs	Placebo [*n* = 137] 19.9 PYs	UPA 45 mg QD [*n* = 247] 38.1 PYs	Placebo [*n* = 139] 82.9 PYs	UPA 15 mg QD [*n* = 141] 119.5 PYs	UPA 30 mg QD [*n* = 149] 133.0 PYs	Placebo [*n* = 86] 45.7 PYs	UPA 15 mg QD [*n* = 86] 65.5 PYs	UPA 30 mg QD [*n* = 84] 72.5 PYs
Any TEAE	213 [736.4] [637.5, 835.3]	499 [786.2] [717.3, 855.2]	154 [773.0] [650.9, 895.0]	329 [864.1] [770.8, 957.5]	353 [425.8] [381.4, 470.2]	371 [310.4] [278.8, 342.0]	411 [309.1] [279.2, 339.0]	263 [574.9] [505.4, 644.4]	190 [289.9] [248.6, 331.1]	211 [290.9] [251.6, 330.1]
Serious TEAE	15 [51.9] [25.6, 78.1]	9 [14.2] [4.9, 23.4]	11 [55.2] [22.6, 87.8]	10 [26.3] [10.0, 42.5]	16 [19.3] [9.8, 28.8]	13 [10.9] [5.0, 16.8]	14 [10.5] [5.0, 16.0]	11 [24.0] [9.8, 38.3]	6 [9.2] [1.8, 16.5]	5 [6.9] [0.9, 12.9]
TEAE leading to discontinuation	15 [51.9] [25.6, 78.1]	12 [18.9] [8.2, 29.6]	15 [75.3] [37.2, 113.4]	5 [13.1] [1.6, 24.6]	16 [19.3] [9.8, 28.8]	7 [5.9] [1.5, 10.2]	15 [11.3] [5.6, 17.0]	10 [21.9] [8.3, 35.4]	4 [6.1] [0.1, 12.1]	4 [5.5] [0.1, 10.9]
Death^a^	0	0	0	0	0	0	0	0	0	0
AESI										
Serious infection	3 [10.4] [0.0, 22.1]	2 [3.2] [0.0, 7.5]	1 [5.0] [0.0, 14.9]	5 [13.1] [1.6, 24.6]	5 [6.0] [0.7, 11.3]	7 [5.9] [1.5, 10.2]	6 [4.5] [0.9, 8.1]	3 [6.6] [0.0, 14.0]	2 [3.1] [0.0, 7.3]	1 [1.4] [0.0, 4.1]
Opportunistic infection [excluding TB and herpes zoster]	0	0	0	3 [7.9] [0.0, 16.8]	0	2 [1.7] [0.0, 4.0]	2 [1.5] [0.0, 3.6]	2 [4.4] [0.0, 10.4]	0	0
Active TB	0	0	0	0	0	0	0	0	0	0
Herpes zoster	0	2 [3.2] [0.0, 7.5]	0	2 [5.3] [0.0, 12.5]	0	6 [5.0] [1.0, 9.0]	11 [8.3] [3.4, 13.2]	0	4 [6.1] [0.1, 12.1]	5 [6.9] [0.9, 12.9]
Neutropenia	1 [3.5] [0.0, 10.2]	32 [50.4] [33.0, 67.9]	0	1 [2.6] [0.0, 7.8]	7 [8.4] [2.2, 14.7]	9 [7.5] [2.6, 12.5]	16 [12.0] [6.1, 17.9]	0	1 [1.5] [0.0, 4.5]	3 [4.1] [0.0, 8.8]
CPK elevation	4 [13.8] [0.3, 27.4]	27 [42.5] [26.5, 58.6]	1 [5.0] [0.0, 14.9]	9 [23.6] [8.2, 39.1]	3 [3.6] [0.0, 7.7]	8 [6.7] [2.1, 11.3]	17 [12.8] [6.7, 18.9]	2 [4.4] [0.0, 10.4]	6 [9.2] [1.8, 16.5]	5 [6.9] [0.9, 12.9]
Any possible malignancy	0	2 [3.2] [0.0, 7.5]	1 [5.0] [0.0, 14.9]	0	1 [1.2] [0.0, 3.6]	1 [0.8] [0.0, 2.5]	1 [0.8] [0.0, 2.2]	1 [2.2] [0.0, 6.5]	0	4 [5.5] [0.1, 10.9]
Any malignancy	0	0	0	0	1 [1.2] [0.0, 3.6]	1 [0.8] [0.0, 2.5]	1 [0.8] [0.0, 2.2]	0	0	4 [5.5] [0.1, 10.9]
Malignancy [excluding NMSC]	0	0	0	0	1 [1.2] [0.0, 3.6]	1 [0.8] [0.0, 2.5]	1 [0.8] [0.0, 2.2]	0	0	1 [1.4] [0.0, 4.1]
NMSC	0	0	0	0	0	0	0	0	0	3 [4.1] [0.0, 8.8]
Lymphoma	0	0	0	0	0	0	0	0	0	0
Renal dysfunction	0	0	0	0	0	0	1 [0.8] [0.0, 2.2]	1 [2.2] [0.0, 6.5]	1 [1.5] [0.0, 4.5]	0
Hepatic disorder	13 [44.9] [20.5, 69.4]	16 [25.2] [12.9, 37.6]	2 [10.0] [0.0, 24.0]	13 [34.1] [15.6, 52.7]	6 [7.2] [1.4, 13.0]	22 [18.4] [10.7, 26.1]	15 [11.3] [5.6, 17.0]	1 [2.2] [0.0, 6.5]	10 [15.3] [5.8, 24.7]	3 [4.1] [0.0, 8.8]
Anaemia	14 [48.4] [23.0, 73.8]	11 [17.3] [7.1, 27.6]	5 [25.1] [3.1, 47.1]	16 [42.0] [21.4, 62.6]	10 [12.1] [4.6, 19.5]	10 [8.4] [3.2, 13.6]	7 [5.3] [1.4, 9.2]	8 [17.5] [5.4, 29.6]	2 [3.1] [0.0, 7.3]	3 [4.1] [0.0, 8.8]
Lymphopenia	2 [3.5] [0.0, 16.5]	9 [14.2] [4.9, 23.4]	1 [5.0] [0.0, 14.9]	11 [28.9] [11.8, 46.0]	4 [4.8] [0.1, 9.6]	9 [7.5] [2.6, 12.5]	3 [2.3] [0.0, 4.8]	0	0	2 [2.8] [0.0, 6.6]
Adjudicated GI perforation	1 [3.5] [0.0, 10.2]	0	0	0	2 [2.4] [0.0, 5.8]	0	0	0	0	0
Adjudicated MACE^b^^,^^c^	0	0	0	0	1 [1.2] [0.0, 3.6]	0	0	0	0	1 [1.4] [0.0, 4.1]
Adjudicated VTE^c^^,^^d^	1 [3.5] [0.0, 10.2]	0	0	0	0	0	1 [0.8] [0.0, 2.2]	0	0	1 [1.4] [0.0, 4.1]

Safety was assessed in all patients who were randomised and received at least one dose of study drug [placebo or UPA 45 mg QD] during the 8-week induction period; for maintenance, this was defined as the UPA 45 mg QD 8-week induction responders who were enrolled per protocol for 44- or 52-week maintenance therapy and received at least one dose of study drug [placebo, UPA 15 mg QD, or UPA 30 mg QD].

^a^Includes treatment-emergent and non-treatment-emergent deaths.

^b^Defined as cardiovascular death, non-fatal myocardial infarction, and non-fatal stroke.

^c^All UPA-treated patients who had an adjudicated VTE or MACE had one or more known risk factors.

^d^Defined as deep vein thrombosis and pulmonary embolism [fatal and non-fatal].

AESI, adverse event of special interest; CI, confidence interval; CPK, creatine phosphokinase; CS, corticosteroids; GI, gastrointestinal; ITT, intention to treat; MACE, major adverse cardiovascular event; NMSC, non-melanoma skin cancer; PY, patient-year; QD, once daily; TB, tuberculosis; TEAE, treatment-emergent adverse event; UPA, upadacitinib; VTE, venous thromboembolic event.

During the maintenance period [Table 3], rates of serious infection were numerically lower in patients with CS use at induction baseline compared with patients with no CS use at induction baseline for both UPA doses [EAER (95% CI): 3.1 (0.0, 7.3) vs 5.9 (1.5, 10.2) E/100 PYs, respectively, for UPA 15 mg QD; and 1.4 (0.0, 4.1) vs 4.5 (0.9, 8.1) E/100 PYs, respectively, for UPA 30 mg QD]. No consistent pattern was observed for herpes zoster between the two UPA doses; the EAER [95% CI] for UPA 15 mg QD was 6.1 [0.1, 12.1] E/100 PYs in patients with CS use at induction baseline and 5.0 [1.0, 9.0] E/100 PYs in patients with no CS use; EAERs for UPA 30 mg QD were 6.9 [0.9, 12.9] vs 8.3 [3.4, 13.2] E/100 PYs, respectively. Higher rates of neutropenia continued to be observed in patients receiving UPA treatment without CS use at induction baseline vs those with CS use. There was one malignancy [excluding NMSC] event in each UPA treatment group without CS use at induction baseline, and one event in the UPA 30 mg QD group with CS use. There were two adjudicated GI perforations reported in the PBO group with no CS use at induction baseline; no adjudicated GI perforation was observed in either UPA group. There was one adjudicated MACE in the PBO group with no CS use at induction baseline and one event in the UPA 30 mg QD group with CS use. For adjudicated VTE, there was one event each in the UPA 30 mg QD groups with and without CS use at induction baseline. EAIRs [*n*/100 PYs] of AESIs by CS use at induction baseline in the safety populations are reported in Supplementary Table 1.

## 4. Discussion

This post hoc analysis of patients with moderately to severely active UC found no apparent difference in the proportions of patients achieving clinical remission per Adapted Mayo Score after induction and maintenance therapy with UPA with or without CS use at induction baseline. The proportion of patients who achieved clinical remission at Week 52 and who had been CS-free for 90 or 183 days immediately before Week 52 was similar in patients with and without CS use at induction baseline. During induction, CS use was associated with numerically higher rates of any TEAE, serious TEAEs, and serious infections compared with no CS use. These risk differences were no longer seen during the maintenance study after withdrawal of CS use. Therefore, withdrawal of CS may be possible during UPA treatment without affecting the achievement of long-term clinical remission, while potentially reducing the safety risks associated with CS use during early stages of UPA exposure.

Given the risks associated with long-term CS use, such as increased mortality, infections, osteoporosis, fractures, and glaucoma,^4,5^ reducing CS use and dependence without impacting on clinical outcomes is an important treatment goal for patients with UC. In this analysis, UPA demonstrated similar proportions of patients achieving clinical remission per Adapted Mayo Score after induction and maintenance treatment, regardless of CS use at induction baseline. Furthermore, a numerically higher proportion of patients with CS use at induction baseline treated with UPA remained CS-free for the entire maintenance period after the 8-week protocol-specified tapering than PBO, and the proportions of patients who were CS-free at each maintenance time point were higher with both UPA doses vs PBO, with a numerically higher proportion seen throughout maintenance with UPA 30 mg QD than UPA 15 mg QD. In a separate analysis of the Phase 3 UPA trials, UPA was found to relieve symptoms including stool frequency, rectal bleeding, abdominal pain, and bowel urgency as early as Day 1 of induction.^15^ Taken together, these results suggest the potential of UPA as an efficacious, steroid-sparing treatment that may allow patients to reduce CS use and associated risks, while still achieving rapid symptomatic control, often highly rated by patients and clinicians.^15^

Among the pre-specified TEAEs of special interest assessed, rates of serious infection, anaemia, and lymphopenia were numerically higher during the induction study with UPA 45 mg QD with CS use than without at induction baseline, but, in the maintenance study after CS use was tapered, similar rates of these events with both UPA doses were observed with and without CS use at induction baseline. Increased rate of infection is a known risk with CS use,^17^ and CS have been found to decrease the number of lymphocytes in blood,^18^ which may account for the increased rates observed during induction. In contrast, rates of neutropenia during induction and maintenance were numerically lower with UPA treatment and baseline CS use than without CS use. Given the known effect of CS to increase the white blood cell count [predominantly neutrophils], the lower rate of neutropenia observed with CS use may be expected, at least during the induction period in which CS dose was unchanged. The reasons behind the differences in anaemia and elevated CPK levels are not clear, but may be confounded by the lack of randomisation by CS use and should be interpreted with respect to this.

Limitations of this analysis include: [i] that patients were stratified, but not randomised, by CS use, meaning it is not possible to determine the impact of CS use during induction on the efficacy and safety of UPA vs no CS use with certainty; [ii] the ideal comparator group of CS-dependent patients who stopped CS at the start of induction [or tapered quickly] was not available; [iii] there were relatively few patients with CS use at induction baseline, although this analysis represents all patients with a clinical response at Week 8 from two large Phase 3 trials and a Phase 2b trial; [iv] stratification was performed by CS use for any indication, rather than explicitly for UC, although the numbers of patients with non-UC indications are expected to be negligible; [v] although patients who exceeded baseline CS dose were considered non-responders for efficacy assessments, these patients were assessed for safety. This means that the safety data during maintenance only assessed the impact of CS use at induction baseline, but did not account for patients re-initiating CS during maintenance; and [vi] further evidence is needed on the long-term, CS-sparing potential of UPA beyond 52 weeks, although there was no apparent reduction in the proportion of patients who remained CS-free over time up to 52 weeks with either UPA maintenance dose.

This post hoc analysis provides evidence in patients with moderately to severely active UC that CS use during induction does not appear to be associated with any difference in achievement of clinical remission after induction or maintenance with UPA. However, patients with CS use receiving UPA during induction had numerically higher rates of TEAEs, serious TEAEs, and serious infections during induction treatment than patients without CS use. Once CS use was withdrawn during maintenance, there were no differences in these rates at Week 52. These results suggest that UPA allows disease control in the absence of CS in this population, and provides a steroid-sparing treatment option that reduces the risk of safety concerns associated with CS.

## Supplementary Material

jjad190_suppl_Supplementary_Material

## Data Availability

AbbVie is committed to responsible data sharing regarding the clinical trials we sponsor. This includes access to anonymised, individual, and trial-level data [analysis datasets], as well as other information [eg, protocols, clinical study reports, or analysis plans], as long as the trials are not part of an ongoing or planned regulatory submission. This includes requests for clinical trial data for unlicensed products and indications. These clinical trial data can be requested by any qualified researchers who engage in rigorous, independent, scientific research, and will be provided following review and approval of a research proposal, Statistical Analysis Plan [SAP], and execution of a Data Sharing Agreement [DSA]. Data requests can be submitted at any time after approval in the USA and Europe and after acceptance of this manuscript for publication. The data will be accessible for 12 months, with possible extensions considered. For more information on the process or to submit a request, visit the following link: [https://www.abbvieclinicaltrials.com/hcp/data-sharing].
