# Supplementary material for: Impact of Baseline Corticosteroid Use on the Efficacy and Safety of Upadacitinib in Patients with Ulcerative Colitis: A Post Hoc Analysis of the Phase 3 Clinical Trial Programme
Source: J Crohns Colitis. 2023 Nov 6;18(5):695–707. doi: 10.1093/ecco-jcc/jjad190 (PMC11140624; doi:10.1093/ecco-jcc/jjad190)
Supplement: jjad190_suppl_Supplementary_Material [file jjad190_suppl_supplementary_material.docx]

# Supplementary Materials

# Supplementary Table 1. EAIRs of AESI by CS use at induction baseline in the safety populations.

|  | Pooled U-ACCOMPLISH and U-ACHIEVE Induction | | | | U-ACHIEVE Maintenance | | | | | |
| --- | --- | --- | --- | --- | --- | --- | --- | --- | --- | --- |
| *n*/PYs [*n*/100 PYs]  [95% CI] | **No CS use at induction baseline** | | **CS use at induction baseline** | | **No CS use at induction baseline** | | | **CS use at induction baseline** | | |
|  | **Placebo  *n* = 332**  **[*n* = 195]**  **28.9 PYs** | **UPA 45 mg QD**  ***n* = 663**  **[*n* = 416]**  **63.5 PYs** | **Placebo  *n* = 332**  **[*n* = 137]**  **19.9 PYs** | **UPA 45 mg QD**  ***n* = 663**  **[*n* = 247]**  **38.1 PYs** | **Placebo  *n* = 225**  **[*n* = 139]**  **82.9 PYs** | **UPA 15 mg QD**  ***n* = 227**  **[*n* = 141]**  **119.5 PYs** | **UPA 30 mg QD**  ***n* = 233**  **[*n* = 149]**  **133.0 PYs** | **Placebo  *n* = 225**  **[*n* = 86]**  **45.7 PYs** | **UPA 15 mg QD**  ***n* = 227**  **[*n* = 86]**  **65.5 PYs** | **UPA 30 mg QD**  ***n* = 233**  **[*n* = 84]**  **72.5 PYs** |
| Serious infections | 2/28.9 [6.9]  [0.8, 25.0] | 2/63.3 [3.2]  [0.4, 11.4] | 1/19.9 [5.0]  [0.1, 28.0] | 5/37.8 [13.2]  [4.3, 30.8] | 5/81.1 [6.2]  [2.0, 14.4] | 7/116.7 [6.0]  [2.4, 12.4] | 6/132.6 [4.5]  [1.7, 9.8] | 3/45.3 [6.6]  [1.4, 19.4] | 1/64.5 [1.5]  [0.0, 8.6] | 1/71.7 [1.4]  [0.0, 7.8] |
| Opportunistic infection [excluding TB and herpes zoster] | 0/28.9 [0.0]  [0.0, 12.8] | 0/63.5 [0.0]  [0.0, 5.8] | 0/19.9 [0.0]  [0.0, 18.5] | 3/38.0 [7.9]  [1.6, 23.1] | 0/82.9 [0.0]  [0.0, 4.4] | 2/119.2 [1.7]  [0.2, 6.1] | 2/132.9 [1.5]  [0.2, 5.4] | 2/44.8 [4.5]  [0.5, 16.1] | 0/65.5 [0.0]  [0.0, 5.6] | 0/72.5 [0.0]  [0.0, 5.1] |
| Active TB | 0/28.9 [0.0]  [0.0, 12.8] | 0/63.5 [0.0]  [0.0, 5.8] | 0/19.9 [0.0]  [0.0, 18.5] | 0/38.1 [0.0]  [0.0, 9.7] | 0/82.9 [0.0]  [0.0, 4.4] | 0/119.5 [0.0]  [0.0, 3.1] | 0/133.0 [0.0]  [0.0, 2.8] | 0/45.7 [0.0]  [0.0, 8.1] | 0/65.5 [0.0]  [0.0, 5.6] | 0/72.5 [0.0]  [0.0, 5.1] |
| Herpes zoster | 0/28.9 [0.0]  [0.0, 12.8] | 2/63.5 [3.2]  [0.4, 11.4] | 0/19.9 [0.0]  [0.0, 18.5] | 1/38.1 [2.6]  [0.1, 14.6] | 0/82.9 [0.0]  [0.0, 4.4] | 6/117.5 [5.1]  [1.9, 11.1] | 10/129.0 [7.8]  [3.7, 14.3] | 0/45.7 [0.0]  [0.0, 8.1] | 4/63.7 [6.3]  [1.7, 16.1] | 4/70.9 [5.6]  [1.5, 14.4] |
| Neutropenia | 1/28.9 [3.5]  [0.1, 19.3] | 30/61.2 [49.0]  [33.1, 70.0] | 0/19.9 [0.0]  [0.0, 18.5] | 1/38.0 [2.6]  [0.1, 14.7] | 5/80.1 [6.2]  [2.0, 14.6] | 5/115.7 [4.3]  [1.4, 10.1] | 12/124.9 [9.6]  [5.0, 16.8] | 0/45.7 [0.0]  [0.0, 8.1] | 1/64.9 [1.5]  [0.0, 8.6] | 3/70.7 [4.2]  [0.9, 12.4] |
| CPK elevation | 4/28.6 [14.0]  [3.8, 35.8] | 24/61.5 [39.0]  [25.0, 58.1] | 1/19.8 [5.1]  [0.1, 28.2] | 8/37.4 [21.4]  [9.2, 42.1] | 3/82.4 [3.6]  [0.8, 10.6] | 7/114.7 [6.1]  [2.5, 12.6] | 15/123.5 [12.1]  [6.8, 20.0] | 2/44.4 [4.5]  [0.5, 16.3] | 6/62.3 [9.6]  [3.5, 21.0] | 4/69.9 [5.7]  [1.6, 14.7] |
| Any possible malignancy | 0/28.9 [0.0]  [0.0, 12.8] | 2/63.4 [3.2]  [0.4, 11.4] | 1/19.9 [5.0]  [0.1, 28.1] | 0/38.1 [0.0]  [0.0, 9.7] | 1/82.9 [1.2]  [0.0, 6.7] | 1/119.5 [0.8]  [0.0, 4.7] | 1/133.0 [0.8]  [0.0, 4.2] | 1/45.6 [2.2]  [0.1, 12.2] | 0/65.5 [0.0]  [0.0, 5.6] | 4/71.4 [5.6]  [1.5, 14.4] |
| Any malignancy | 0/28.9 [0.0]  [0.0, 12.8] | 0/63.5 [0.0]  [0.0, 5.8] | 0/19.9 [0.0]  [0.0, 18.5] | 0/38.1 [0.0]  [0.0, 9.7] | 1/82.9 [1.2]  [0.0, 6.7] | 1/119.5 [0.8]  [0.0, 4.7] | 1/133.0 [0.8]  [0.0, 4.2] | 0/45.7 [0.0]  [0.0, 8.1] | 0/65.5 [0.0]  [0.0, 5.6] | 4/71.4 [5.6]  [1.5, 14.4] |
| Malignancy [excluding NMSC] | 0/28.9 [0.0]  [0.0, 12.8] | 0/63.5 [0.0]  [0.0, 5.8] | 0/19.9 [0.0]  [0.0, 18.5] | 0/38.1 [0.0]  [0.0, 9.7] | 1/82.9 [1.2]  [0.0, 6.7] | 1/119.5 [0.8]  [0.0, 4.7] | 1/133.0 [0.8]  [0.0, 4.2] | 0/45.7 [0.0]  [0.0, 8.1] | 0/65.5 [0.0]  [0.0, 5.6] | 1/72.5 [1.4]  [0.0, 7.7] |
| NMSC | 0/28.9 [0.0]  [0.0, 12.8] | 0/63.5 [0.0]  [0.0, 5.8] | 0/19.9 [0.0]  [0.0, 18.5] | 0/38.1 [0.0]  [0.0, 9.7] | 0/82.9 [0.0]  [0.0, 4.4] | 0/119.5 [0.0]  [0.0, 3.1] | 0/133.0 [0.0]  [0.0, 2.8] | 0/45.7 [0.0]  [0.0, 8.1] | 0/65.5 [0.0]  [0.0, 5.6] | 3/71.4 [4.2]  [0.9, 12.3] |
| Lymphoma | 0/28.9 [0.0]  [0.0, 12.8] | 0/63.5 [0.0]  [0.0, 5.8] | 0/19.9 [0.0]  [0.0, 18.5] | 0/38.1 [0.0]  [0.0, 9.7] | 0/82.9 [0.0]  [0.0, 4.4] | 0/119.5 [0.0]  [0.0, 3.1] | 0/133.0 [0.0]  [0.0, 2.8] | 0/45.7 [0.0]  [0.0, 8.1] | 0/65.5 [0.0]  [0.0, 5.6] | 0/72.5 [0.0]  [0.0, 5.1] |
| Renal dysfunction | 0/28.9 [0.0]  [0.0, 12.8] | 0/63.5 [0.0]  [0.0, 5.8] | 0/19.9 [0.0]  [0.0, 18.5] | 0/38.1 [0.0]  [0.0, 9.7] | 0/82.9 [0.0]  [0.0, 4.4] | 0/119.5 [0.0]  [0.0, 3.1] | 1/133.0 [0.8]  [0.0, 4.2] | 1/45.8 [2.2]  [0.1, 12.2] | 1/65.5 [1.5]  [0.0, 8.5] | 0/72.5 [0.0]  [0.0, 5.1] |
| Hepatic disorder | 6/28.3 [21.2]  [7.8, 46.1] | 11/62.6 [17.6]  [8.8, 31.5] | 2/19.7 [10.2]  [1.2, 36.7] | 8/37.5 [21.3]  [9.2, 42.0] | 4/81.3 [4.9]  [1.3, 12.6] | 10/112.4 [8.9]  [4.3, 16.4] | 9/128.7 [7.0]  [3.2, 13.3] | 1/44.8 [2.2]  [0.1, 12.4] | 7/62.5 [11.2]  [4.5, 23.1] | 2/71.6 [2.8]  [0.3, 10.1] |
| Anaemia | 13/27.8 [46.7]  [24.9, 79.9] | 11/62.3 [17.7]  [8.8, 31.6] | 5/19.7 [25.3]  [8.2, 59.1] | 14/37.0 [37.9]  [20.7, 63.6] | 8/79.4 [10.1]  [4.4, 19.9] | 9/113.0 [8.0]  [3.6, 15.1] | 6/130.1 [4.6]  [1.7, 10.0] | 6/42.5 [14.1]  [5.2, 30.7] | 2/64.6 [3.1]  [0.4, 11.2] | 3/70.7 [4.2]  [0.9, 12.4] |
| Lymphopenia | 1/28.8 [3.5]  [0.1, 19.4] | 6/63.0 [9.5]  [3.5, 20.7] | 1/19.9 [5.0]  [0.1, 28.0] | 10/37.2 [26.9]  [12.9, 49.5] | 3/81.1 [3.7]  [0.8, 10.8] | 6/114.8 [5.2]  [1.9, 11.4] | 2/132.2 [1.5]  [0.2, 5.5] | 0/45.7 [0.0]  [0.0, 8.1] | 0/65.5 [0.0]  [0.0, 5.6] | 2/71.8 [2.8]  [0.3, 10.1] |
| Adjudicated GI perforation | 1/28.9 [3.5]  [0.1, 19.3] | 0/63.5 [0.0]  [0.0, 5.8] | 0/19.9 [0.0]  [0.0, 18.5] | 0/38.1 [0.0]  [0.0, 9.7] | 1/82.9 [1.2]  [0.0, 6.7] | 0/119.5 [0.0]  [0.0, 3.1] | 0/133.0 [0.0]  [0.0, 2.8] | 0/45.7 [0.0]  [0.0, 8.1] | 0/65.5 [0.0]  [0.0, 5.6] | 0/72.5 [0.0]  [0.0, 5.1] |
| Adjudicated MACE^a,b^ | 0/28.9 [0.0]  [0.0, 12.8] | 0/63.5 [0.0]  [0.0, 5.8] | 0/19.9 [0.0]  [0.0, 18.5] | 0/38.1 [0.0]  [0.0, 9.7] | 1/82.9 [1.2]  [0.0, 6.7] | 0/119.5 [0.0]  [0.0, 3.1] | 0/133.0 [0.0]  [0.0, 2.8] | 0/45.7 [0.0]  [0.0, 8.1] | 0/65.5 [0.0]  [0.0, 5.6] | 1/71.8 [1.4]  [0.0, 7.8] |
| Adjudicated VTE^b,c^ | 1/28.9 [3.5]  [0.1, 19.3] | 0/63.5 [0.0]  [0.0, 5.8] | 0/19.9 [0.0]  [0.0, 18.5] | 0/38.1 [0.0]  [0.0, 9.7] | 0/82.9 [0.0]  [0.0, 4.4] | 0/119.5 [0.0]  [0.0, 3.1] | 1/133.0 [0.8]  [0.0, 4.2] | 0/45.7 [0.0]  [0.0, 8.1] | 0/65.5 [0.0]  [0.0, 5.6] | 1/72.4 [1.4]  [0.0, 7.7] |

Safety was assessed in all patients who were randomised and received at least one dose of study drug [PBO or UPA 45 mg QD] during the 8-week induction period; for maintenance, this was defined as the UPA 45 mg QD 8-week induction responders who were enrolled per protocol for 44- or 52-week maintenance therapy and received at least one dose of study drug [placebo, UPA 15 mg QD, or UPA 30 mg QD].

^a^Defined as cardiovascular death, non-fatal myocardial infarction, and non-fatal stroke.

^b^All UPA-treated patients who had an adjudicated VTE or MACE had one or more known risk factors.

^c^Defined as deep vein thrombosis and pulmonary embolism [fatal and non-fatal].

AESI, adverse event of special interest; CI, confidence interval; CPK, creatine phosphokinase; CS, corticosteroid; EAIR, exposure-adjusted event rate; GI, gastrointestinal; ITT, intention to treat; MACE, major adverse cardiovascular event; n, number of patients; NMSC, non-melanoma skin cancer; PBO, placebo; PY, patient-year; QD, once daily; TB, tuberculosis; TEAE, treatment-emergent adverse event; UPA, upadacitinib; VTE, venous thromboembolic event.

**Supplemental Figure 1.** A sensitivity analysis of the proportion of patients in clinical remission per Adapted Mayo Score at the end of maintenance [Week 52], by CS use at induction baseline^a^


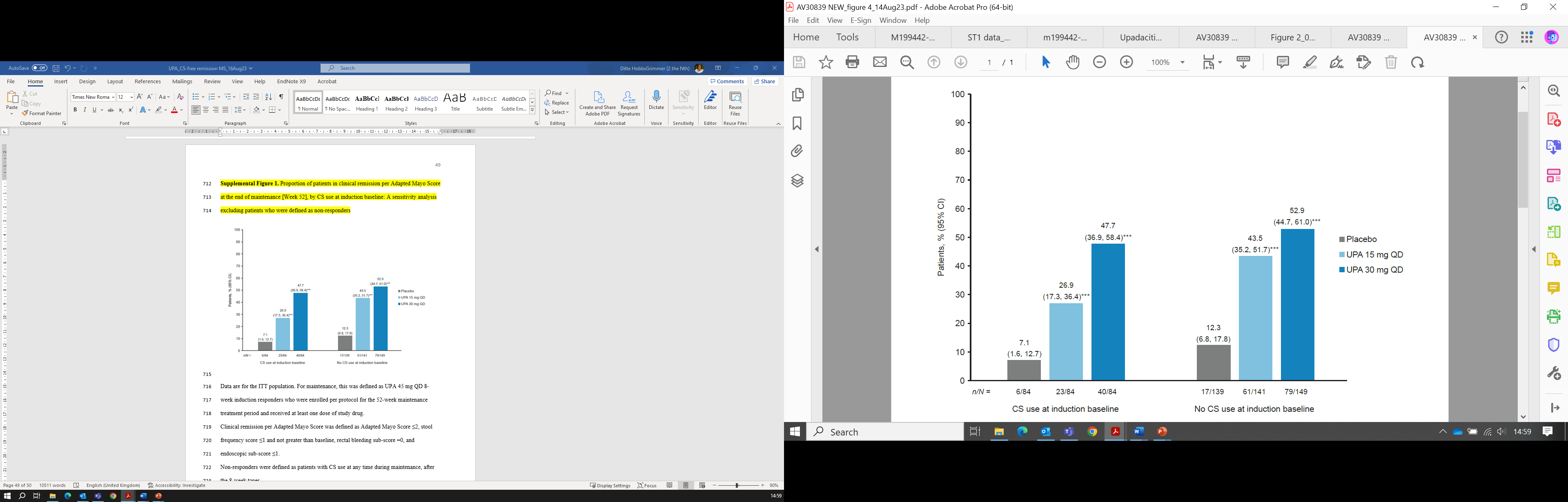


Data are for the ITT population. For maintenance, this was defined as UPA 45 mg QD 8-week induction responders who were enrolled per protocol for the 52-week maintenance treatment period and received at least one dose of study drug.

Clinical remission per Adapted Mayo Score was defined as Adapted Mayo Score ≤2, stool frequency score ≤1 and not greater than baseline, rectal bleeding sub-score =0, and endoscopic sub-score ≤1.

^a^Analysed in the same way as the main analysis, except that any patient who received CS after Week 8 of maintenance was considered a non-responder.

***Nominal *p* <0.001 vs placebo.

CI, confidence interval; CS, corticosteroid; ITT, intention to treat; QD, once daily; UPA, upadacitinib.

**Supplemental Figure 2.** A sensitivity analysis of Partial Mayo Score from Week 0 to Week 52 of maintenance in patients with or without CS use at induction baseline^a^


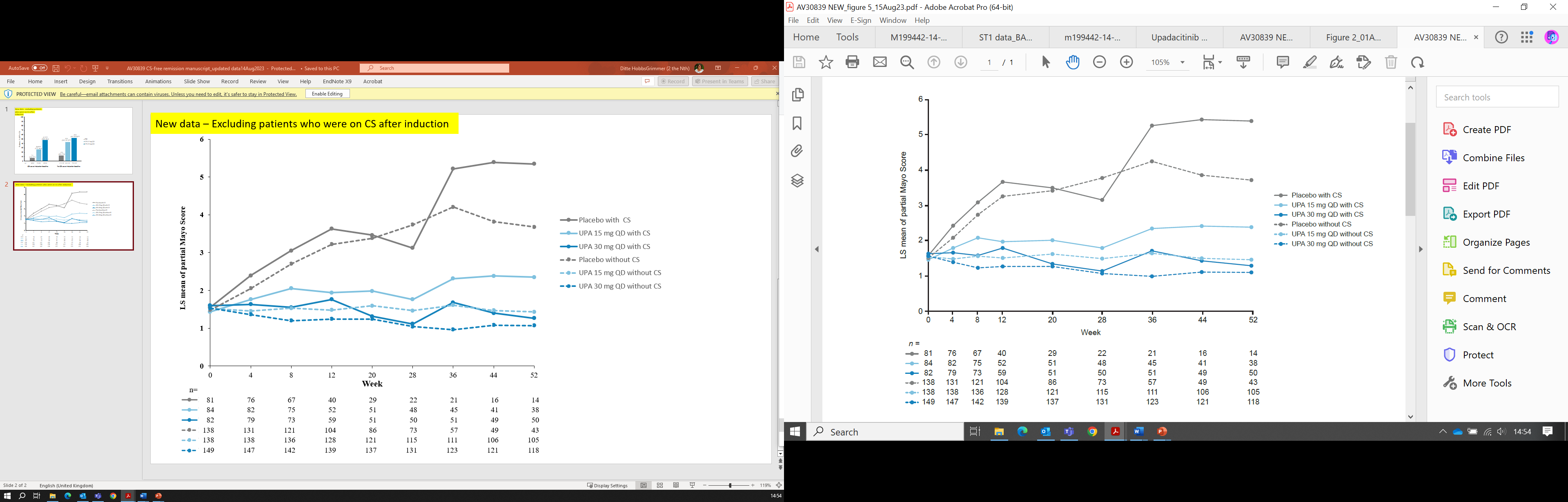
 Data are for the ITT population. For maintenance, this was defined as UPA 45 mg QD 8-week induction responders who were enrolled per protocol for the 52-week maintenance treatment period and received at least one dose of study drug [placebo, UPA 15 mg QD, or UPA 30 mg QD].

^a^Analysed in the same way as the main analysis, except that if a patient received CS after Week 8, measurements were excluded for that patient from the CS start date onwards.

CS, corticosteroid; ITT, intention to treat; LS, least squares; QD, once daily; UPA, upadacitinib.
